# Sepsis and Immunosenescence in the Elderly Patient: A Review

**DOI:** 10.3389/fmed.2017.00020

**Published:** 2017-02-28

**Authors:** Silvia Martín, Alba Pérez, Cesar Aldecoa

**Affiliations:** ^1^Anaesthesia and Surgical Critical Care, Hospital Universitario rio Hortega, Valladolid, Spain; ^2^University of Valladolid Medical School, Valladolid, Spain

**Keywords:** sepsis, immunosenescence, elderly patients, outcome, quality of life

## Abstract

Sepsis is a prevalent, serious medical condition with substantial mortality and a significant consumption of health-care resources. Its incidence has increased around 9% annually in general population over the last years and specially in aged patients group. Several risk factors such as comorbidities, preadmission status, malnutrition, frailty, and an impared function in the immune system called immunosenescence are involved in the higher predisposition to sepsis in the elderly patients. Immunosenescence status consists in a functional impairment in both cell-mediated immunity and humoral immune responses and increases not only the risk for develop sepsis but also lead to more severe presentation of infection and may be is also related with a higher mortality. There is a also a concern about to admit patients in the intensive care units taking into account that the outcome of elderly patients is poorer compared to younger people. Nevertheless, the management of septic elderly patients does not differ substantially from younger people. In addition, the quality of life in septic elderly survivors is also lower than in younger people. But age, as alone factor, should not be used to determine treatment options because the poorer outcomes is thought to be due to the increased comorbidities and frailty in this group of patients.

## Introduction

Sepsis is a frequent condition in critically ill patients and is associated with a substantial increase in health-care resources consumption. The number of cases of sepsis rose by an average of 9% per year. The increase in the incidence of sepsis has been also accompanied by an increase in sepsis-related mortality despite actual decreases in mortality rates among hospitalized patients. On the other hand, the rapidly increased life expectancy in developed countries makes to understand the different characteristics of severe sepsis in older patients a clinical priority and a challenge for clinicians.

For years, there has been a steady increase in the percentage of people older than 60 years as compared to the total population. In the 1950s, they accounted for only 8% of the population, increasing to 10% in 2000, and estimated to reach 21% by 2050 (1). Over the next few decades, the population aged over 80 years will double. In 2050, octogenarians will account for 9.6% of the total population in Europe and 9% in North America (2). One of the problems arising from population aging is an increased incidence and severity of diseases, such as sepsis both in the forms of community-acquired and hospital-acquired, as compared to the younger counterparts (3).

Older persons are more prone to infections due to the effects of aging, comorbidities, use of invasive devices, and factors associated with institutionalization. It has been shown that around 40–50% of all cases of bacteremia occur in older patients (4, 5). In addition, the incidence and mortality of severe sepsis in older patients has increased progressively to achieve a rate of 26.2 cases per 1,000 persons, with a 38.4% mortality rate in patients older than 85 years (3).

This increment in the incidence of severe sepsis in aging people has been reflected in the demographic changes of bed allocation in intensive care units (ICUs), where, at present, older patients represent around two-thirds of occupations (6). This situation will further increase over the forthcoming years as well as the amount of health-care resources required by these patients.

According to the World Health Organization definition, the segment of elderly people can be divided into young patients (less than 65 years), young elderly (aged 65–85 years), and old elderly (over 85 years) (7). It is well known that old elderly patients (over or 85 years) is a high-risk population due to their frailty and associated morbidity (8).

Moreover, there is a concern about the fate of very old patients admitted to the ICU, taking into account that the outcome of young elderly and old elderly ICU patients is usually worse than that of younger patients independently of the underlying diagnosis. Thus, important questions regarding suitability of ICU admission, treatment, and quality of life in this group of patients still remain unanswered.

In this review, we focus on risk factors, age-related pathophysiologic mechanisms, and immunologic events that impair host antimicrobial defenses in elderly patients with septic shock. We also discuss recent findings regarding the clinical impact, therapeutic strategies, and prognosis in older patients with sepsis.

## Risk Factors

There are several risk factors predisposing elderly patients to an increased incidence of sepsis.

### Comorbidities and Previous Disorders

Comorbidities and previous disorders as renal and pulmonary diseases are usually present in association with an increased susceptibility to sepsis, but these comorbidities alone are not sufficient for causing infection, and other factors, such as treatment with various drugs or recurrent hospitalizations are necessary to compromise immunity in old patients (9).

### Preadmission Status

Preadmission status is as important as comorbidities and has even been shown to be an independent predictor of outcome in the elderly (10). The main alterations in functional status include disuse atrophy from an inactive lifestyle, sarcopenia from accelerated muscle loss, changes in responsiveness to trophic hormones (growth hormones, androgens, and estrogens), neurological alterations, altered cytokine regulation, changes in protein metabolism, and changes in dietary intake (6).

### Malnutrition

Malnutrition is also common in the old patient. The fact that olfactory discrimination is reduced by age causes an impair in different tastes and produces less pleasure for food, contributing to the lack of appetite. The nutritional condition of older people can be influenced by several factors such as little physical activity, few resources, motion problems, social isolation, inadequate diet, chronic conditions, cognitive impairment, mood disorders, teething issues, use of too many medicines, and alcohol or substance abuse (6, 9).

### Drugs

It is necessary to improve our knowledge regarding how aging affects the absorption, hepatic metabolism, and response to drugs. Pharmacokinetics of drugs is altered in older persons, which may result in an increase in potential drug interactions usually due to the high number of medications taken, rather than to age (11).

### Intestinal Microbiota

Gastrointestinal balance is affected by age. Physiologic age-related changes are due to modification in diet, lifestyle, and reduction of functionality of the immune system. Therefore, these impairments in intestinal microbiota could be principal actors of distinctive conditions of the elderly patient, such as frailty, immunosenescence, metabolic syndrome, diabetes, and sarcopenia. The gastrointestinal tract contains the most complex bacterial ecosystem of the human body and the genome of this intestinal microbiota (microbiome) is made up of more than 100 times the number of genes in the human genome. In summary, changes related to age in intestinal microbiota contribute to the pathophysiologic processes causing malnutrition, empowering inflammatory status, and susceptibility to infection. However, the gut microbiota composition of elderly people is still uncertain, so we need more studies to complete it, as well as more research of new possible treatment strategies such as pro/prebiotics, which could be a useful support in individual nutritional strategies to improve or preserve elderly population’s health (12).

### Immunosenescence

The innate immune system is damaged, so it is easier for pathogens to access the organism. Indeed, there is enough evidence of T- and B-cell dysfunction in aging people.

## Pathophysiology

Aging is unavoidable in humans; it is a polygenic process determined genetically on the one hand and intimately related to exogenous factors influencing each of the individuals throughout their life (13, 14). The steady accumulation of somatic mutations during an individual’s life results in a decreased capacity of cell regeneration, cell repair, and an altered function of the immune system.

The immune cells are constantly renewed from the hematopoietic stem cells, and in elderly subjects, both the proliferative capacity and the number of these immune cells are decreased due to progressive telomere shortening, resulting in an immune dysfunction over the years, which is known as immunosenescence (15).

This state of immunosenescence predisposes to the risk for the development of sepsis and also causes alterations in the body’s response leading to a more severe presentation of infection.

### Immunosenescence in the Elderly

There are functional impairments in both cell-mediated immunity and humoral immune responses with age. The thymus, which is involved in adaptive cell-mediated immunity, atrophies with age and by 60 years causes less activity in the T-cell repertoire from naïve T-cells to memory T-cells.

Macrophages present functional alterations, as decreased antigen processing and expression to T-cells, reduced bactericidal activity and altered expression and function of toll-like receptors. Furthermore, others cells involved in innate immunity like neutrophils and natural killer (NK) cells are also impaired causing reduced recognition and destruction of infected cells (13–24) (Table 1).

**Table 1:** **Immunosenescence**.

Innate immunity (15–17, 22, 37, 43, 46)	Decreased function of macrophages (chemotaxis, phagocytosis, apopotosis, TLR expression, and cytokine production)
	Decreased function of neutrophils (chemotaxis, phagocytosis, signal transduction, and apoptosis)
	Decreased function in dendritic cells (antigen presentation, chemotaxis, and endocytosis)
	Decreased in phagocytic capacity
	Decreased sensitivity to IFN and growth hormone
	Decreased production of TNF-α and IL-6
	Increased production of IL-10
	Decreased sensitivity to G-CSF
	Decreased expression of TLRs
	Increased number of NK cells
	Decline in NK cell function
	Circulating inmature neutrophils

T-cells (17, 19, 20, 23–25, 40, 43, 44, 46)	Decreased naïve cells
	Decrease naïve CD4 function
	Decrease naïve CD8 function
	Decreased type 1 cytokine response
	Increased type 2 cytokine response
	Decreased function of mitogen-activated protein kinases

B-cells (17, 18, 44, 46)	Decrease in the number of B-cells
	Reduced antibody affinity
	Decreased response to neoantigens
	Increased level of antibodies

*TLR, toll-like receptor; IFN, interferon; TNF, tumor necrosis factor; IL, interleukin; G-CSF, granulocyte colony-stimulating factor; NK, natural killer*.

#### Effects of Age on Innate Immunity

The main cells of the innate immune system and the soluble mediators (cytokines, hormones, and free radicals) are both well preserved in the elderly, even at extreme ages. However, there are biochemical and cell function alterations facilitating infection. It has been shown that plasma levels of interleukin (IL)-6 and IL-1 and tumor necrosis factor (TNF) are elevated in the elderly population. This represents a state of constant stimulation of the immune system and, therefore, a continuous subclinical inflammatory state, which would explain the progression and development of many pathologic processes in the elderly (15, 16).

This inflammatory cellular microenvironment also causes changes in cell differentiation and behavior of antigen-presenting cells. In fact, IL-10, the function of which is to suppress cell-mediated immunity, is increased in older healthy individuals (23–25). Despite these changes in functionality, normal values of leukocytes, total lymphocytes, and B- and T-lymphocyte subpopulations have been reported in observational studies. Many differences in cell parameters are found among studies, with the largest differences in the CD4/CD8 ratio (26).

A more prolonged proinflammatory response has been generally observed in elderly patients than in young people. A reduction in clearance of microbial pathogens could be involved in this response. In addition, impairment in the attenuation of signaling by counterregulatory cytokines may play a role.

This persistent inflammation could induce an exhaustion of T-cells, which may be associated with a reduction in survival in the elderly probably due to an increase in the susceptibility to secondary infections (27). Among innate immune cells, neutrophils both in the number of these cells and their precursors in the bone marrow are preserved in the elderly (26–32).

By contrast, the number of monocytes and macrophages in peripheral blood in elderly subjects are similar to younger people, but a significant decrease in macrophage precursors in the bone marrow has been found (32). Several studies have shown a decreased function of these cells in relation to age. A reduction of the phagocytic ability of neutrophils has been observed and this low neutrophil response is particularly important in the susceptibility to pathogens, such as *Staphylococcus aureus*, seen in older people (15, 29).

In addition, impairment in superoxide generation and an increase in the apoptosis in neutrophils from elderly patients after antigen stimulation have been observed (33). A reduction of intracellular calcium flux and a decline in glycosylation patterns of intracellular proteins has been found in activated neutrophils from elderly patients (7, 34, 35).

**Figure 1:**
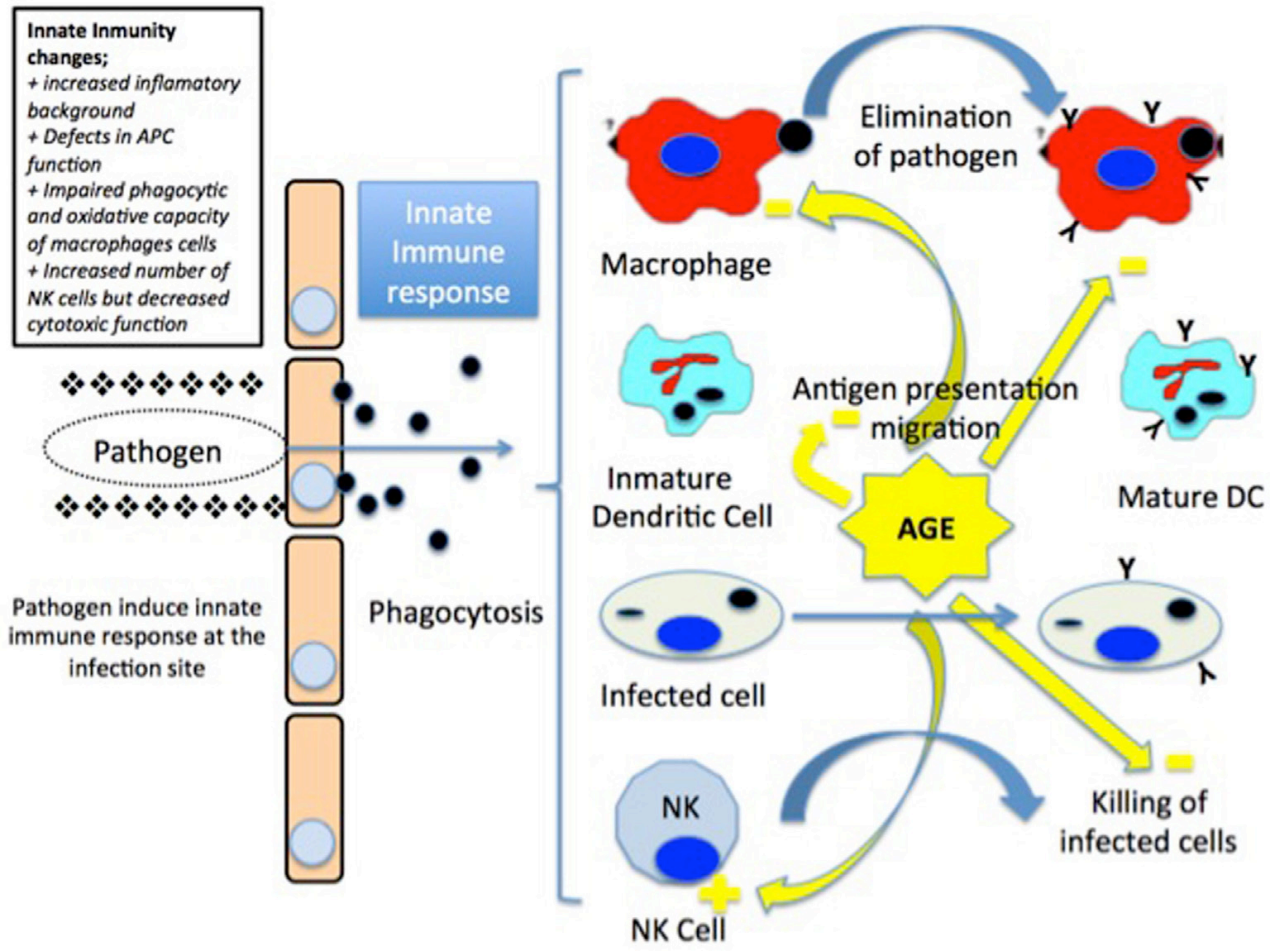
**Invading pathogens induce innate immune responses at the infection point**. The pathogen agent is taken up by macrophages and dendritic cells (DCs). Macrophages present the antigen to the lymph nodes in major histocompatibility complex class II (MHC class II) molecules associated to the secretion of proinflammatory cytokines. On the other side, mature DCs migrate to the lymph node and present both MHC class I and II molecules. Infected cells are eliminated by natural killer (NK) cells [modified from Ref. (15), with permission of John Wiley and Sons].

Moreover, it has been observed a reduction in the macrophages function with a decrease in the levels of the major histocompatibility complex type II (MHC II) molecules, which results in a decreased response of CD4 histocompatibility. Also, there is an increase in the synthesis of prostaglandin E2 that stimulates the production of cytokines by T-lymphocytes and the long-term reconversion of T-lymphocytes type 1 helper into type 2 helper (34, 36) (Figure 1).

On the other hand, NK cells also play an important role in innate immunity. Impairment in elderly have been observed in several studies indicating that the number of NK cells that increases with age may be due to a compensatory response to a relative loss of the lytic activity (33, 36–40). However, their cytotoxic capacity decreases. In fact, it has been observed that NK cells have a decreased production and response to interferon gamma (IFN-γ) and chemokines after stimulation with IL-2 or IL-12 (34, 36, 37).

#### Effects of Age on the Adaptive Immune System

**Figure 2:**
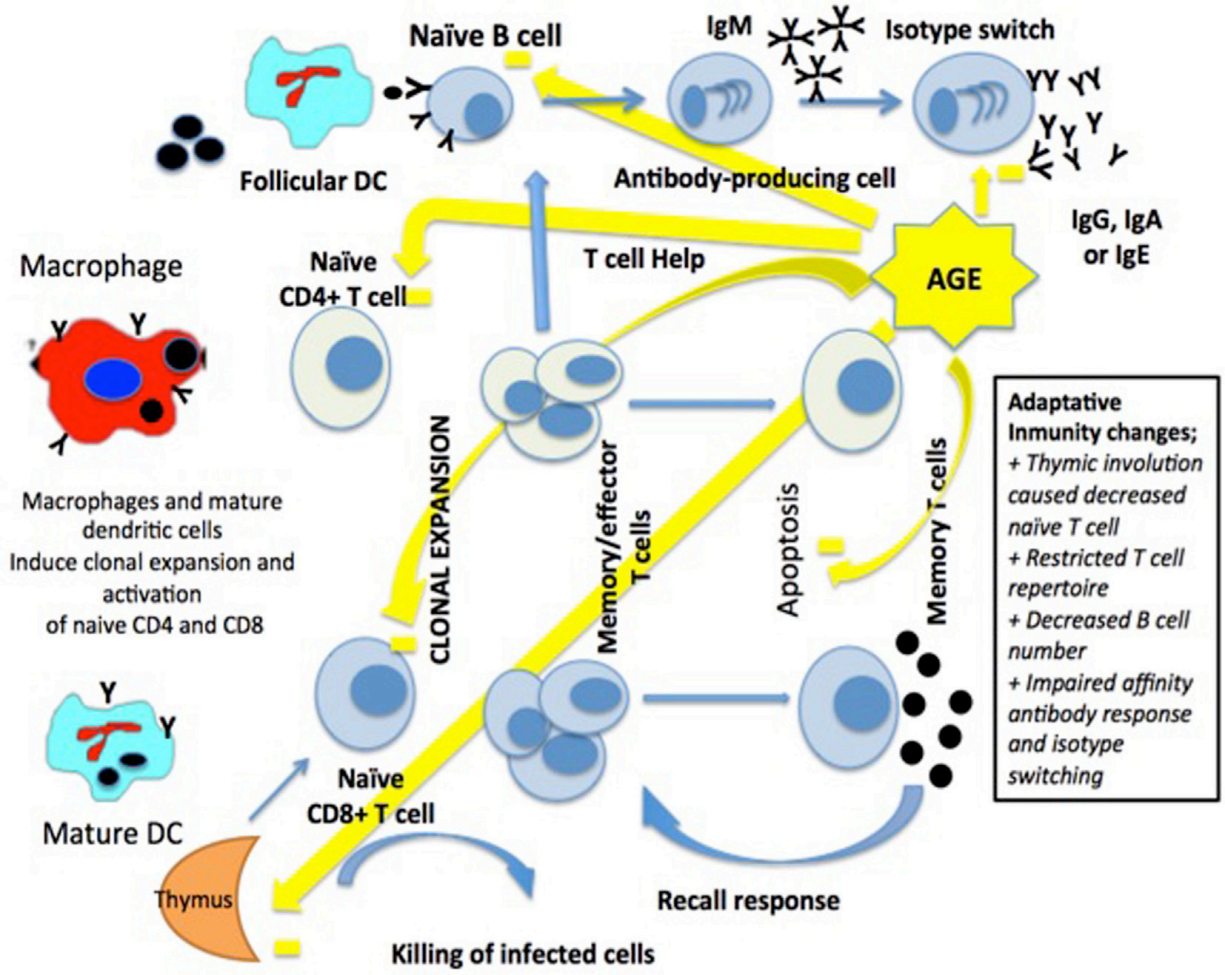
**Macrophages and dendritic cells (DCs) activate a clonal expansion of naïve CD4+ and CD8+ T-cell**. CD4+ T-cell help and the antigen induce the differentiation of naïve B-cells, then into memory B-cells and antibody-secreting cells. Long-term immunity in the blood and lymph nodes is related to T-cells and B-cells [modified from Ref. (15), with permission of John Wiley and Sons].

The adaptive immune system is represented by T- and B-lymphocytes. T-lymphocytes mature in the thymus, and this process occurs mainly in early childhood until involution of the gland by the age of 60. The phenomenon of decreased thymopoiesis produces a dramatic reduction of naïve T-lymphocytes and although levels remain adequate for years, decreases are particularly relevant over 70 years of age (36, 39) (Figure 2).

Thymic involution causes a decrease in the production of CD4 and CD8 lymphocytes leading to a poorer response to neoantigens exposure affecting the adaptive immune system when faced to systemic bacterial infections (40).

One of the reasons for the decrease of the adaptive immune response observed in older people is caused by the accumulation of effector T-cells with functional alterations resulting in a reduction in either cell receptors or IL-2. In addition, an increase of memory T-cells leading to an increase in the production of cytokines has been reported (33).

In older people, there is a state of chronic inflammation caused by an increase of IL-6 and TNF-α levels, resulting in an increased sensitivity of CD4 and CD8 cells to induced apoptosis by TNF-α. Also, there is an inverted CD4/CD8 ratio, which may be associated with an increased mortality in these age groups (38, 41, 42).

The CD4/CD8 ratio is used as a marker of both immunosenescence and immune activation. It has been also considered a mechanism of immune defense and repair. The normal range of the CD4/CD8 ratio is between 0.9 and 1.9 in the HIV-negative population (43). In elderly patients, most studies have shown an increase of CD4/CD8 ratio with age due to CD8 decrease, with a normal range between 1.6 and 2.2 (26). Moreover, in nonagenarian patients, there are differences in comparison to younger elderly, with an increase of CD4/CD8 ratio in an important percentage of patients (44).

In the humoral immune system, significant changes in relation to advanced age are also noticed. Plasma B-cells gradually decrease; however, elevated levels of circulating immunoglobulins especially those derived from polyspecific antibody-producing B lymphocyte cells with low affinity for antigens are seen. Many of these antibodies react as autoimmune antibodies. Moreover, the production of highly specific antibodies produced by B-lymphocytes after contact with T-cells is reduced due to lack of function of such cells (37).

All these defects contribute to reduce the response of the adaptive immune system to pathogens, with an increased risk for developing systemic infections, such as sepsis, and also to an impaired and reduced vaccine response. In order to investigate the effects of aging on humoral immune response, some studies have analyzed Ig heavy-chain transcript sequences in both young and elderly populations before and after vaccination. Elderly patients generally showed a worse and less diversified response than younger people that may indicate the negative effect of age on the functionality of B-cells (45).

Immunosenescence affects both innate and adaptive immunity, and this is reflected in specific T-cell marks such as an inverse CD4/CD8 ratio, loss of naïve T-cells, increased numbers of terminally differentiated T-cells, and a reduction in the function of NK cells, leading to a decreased response to vaccination in the elderly. Accordingly, it is important to develop new strategies (new adjuvants, dose and booster adaptation, and immunization routes) and protocols to improve vaccine responses, making them more effective to prevent infectious diseases and infection-related morbidity and mortality (46).

#### Coagulation

Advanced age is associated with a procoagulable state. Different studies have shown an increase in coagulation factors and thrombin–antithrombin complexes in healthy older patients as compared to healthy younger age subjects. Elevated plasminogen activator inhibitor-type I levels has also been observed. This leads to an increased risk of thrombosis and decreased clearance of circulating fibrin (31, 41).

## Diagnosis

The diagnosis of sepsis is challenging and likely to be missed if not anticipated. Early signs and symptom may go unnoticed, while later clinical presentation can be very severe with very rapid progression to septic shock. In fact, the classical manifestations of systemic inflammatory response syndrome may be minimally present.

In elderly septic patients, febrile response could be blunted in up to 47% of cases, but other unusual or non-specific signs, such as weakness, malaise, delirium, confusion, loss of appetite, falls, or urinary incontinence may be present (41).

In many cases, establishing a prompt and correct diagnosis is further complicated by a lack of cooperation in the frail, dehydrated, debilitated, and cognitively impaired patient, which is a common situation in aging population. It is well known that speed in establishing an accurate diagnosis and implementation of rapid and effective resuscitation treatment are essential factors for optimizing survival rates. However, diagnosing sepsis is not always straightforward, particularly in the elderly patient who often have complex ongoing disease processes.

The optimal diagnosis of infection and sepsis in all patients is based on clinical signs and symptoms. Biologic laboratory markers (biomarkers), such as white blood cell count, C-reactive protein, procalcitonin, cytokine levels, and (to some extent) coagulation markers can potentially be used to assess prognosis and development of organ dysfunction, as well as to guide antimicrobial treatment and evaluation of response.

Recently, new definitions of sepsis aimed to facilitate and accelerate the recognition of patients at risk of developing sepsis have been proposed. Original concepts of sepsis were focused on inflammation response, but new definitions underline the pathobiology mechanisms of the disease. This approach helps stratifying patients at risk of sepsis not only for intensivists but also for other clinicians who may be less familiarized with this type of patients (47).

In this new perspective, sepsis is defined as a life-threatening organ dysfunction caused by a dysregulated host response to infection. We need to identify infected patients most likely to develop sepsis as soon as possible, and sequential organ failure assessment (SOFA) is the score most commonly used in the ICU setting. An elevated SOFA score is associated with an increased risk of mortality. Although this score is not useful for the management of patients, it is a valuable indicator to characterize septic patients. The quick SOFA score based on high respiratory rate (≥22 beats/min), low systolic blood pressure (≤100 mm Hg), or altered mentation (Glasgow coma scale <15) is useful to suspect infection in patients who are at greater risk for a poor outcome (47).

In elderly patients, the most common source of sepsis is the respiratory tract followed by genitourinary infections, but, in general, the sites of infection are similar to patients of other age groups, involving the respiratory, urinary, and gastrointestinal systems as well as the skin and soft tissues (9, 48, 49).

Also, elderly patients are at increased risk of infections caused by multidrug-resistant organisms not only due to immunosenescence but also because of a greater exposure to institutionalization and an increased early use of broad-spectrum antibiotics, which may select more virulent and resistant strains (48, 49).

Some studies found an increased rate of methicillin-resistant *S. aureus* and vancomycin-resistant *Enterococci* as causative organism of sepsis in older patients. Also, the incidence of extended-spectrum β-lactamase producing *Klebsiella* spp. has been found to be higher among patients older than 65 years and younger than 14 years (9, 48, 49).

## Management

The “Surviving Sepsis Campaign: International Guidelines for Management of Sepsis and Septic Shock” should be followed for the general care of elderly patients with severe sepsis and septic shock with the same aim of rapid initial management, including the 3- and 6-h reanimation bundles, and all measures proposed regarding hemodynamic monitoring, mechanical ventilation strategies, infection source control, fluid therapy, and nutrition (6, 48). On the other hand, a few specific considerations for the elderly septic patient should be taken into account.

In the elderly septic patient, it is mandatory to maintain an adequate preload in order to increase the cardiac output. However, aging patients usually have diastolic dysfunction, so that overzealous fluid administration can be problematic. Dobutamine, which can be used to improve tissue perfusion could produce different effects because of a relative resistance observed in the elderly patient and the arrhythmogenic response, in particular, in patients with cardiac ischemic disease (9, 48).

In relation to blood transfusion, a hemoglobin target of 7–9 g/dL, the same than in young adults should be attained. However, we should be alert in patients with low central venous oxygen saturation and ischemic myocardiopathy, which may be frequent in advanced age, because the protocol contradicts the early goal-directed resuscitation that targets a hematocrit of 30%.

The differences in pharmacokinetic and pharmacodynamic parameters such as reduced renal function make us to adjust the dosage of antibiotics as well as to consider an increased frequency of antimicrobial-related adverse effects in the elderly. In addition, older patients may be more susceptible to adverse drug events than younger patients (29, 50, 51).

Levosimendan is a calcium-sensitizing drug with inotropic and vasodilator properties to treat decompensated heart failure (52). Comparing with dobutamine, levosimendan use in patients with septic shock has shown improvements in hemodynamic variables (microcirculatory flow (53), renal, and hepatic function) (54). Results of a meta-analysis support the use of levosimendan in patients with sepsis but only 125 patients in total had been treated (55). However, data regarding the use of levosimendan in the elderly group are scarce. In a recent randomized controlled trial, levosimendan as standard therapy in a septic shock has not shown any improvement in survival or organ dysfunction (56).

The influence of age on the effect of the vasopressor agents has been recently examined in the study of Biston et al. (51) showing that the doses of vasopressor and adrenergic agents were similar independently of age and, therefore, interaction between age and vasopressor agents has no significant effect on outcome.

There is controversy with the use of steroids for septic shock, despite the fact that adrenal insufficiency is common in patients with advanced age.

In the Surviving Sepsis Campaign, due to scarce evidence of the lack of effectiveness and serious adverse effects of steroids, including hyperglycemia, immunosuppression, and exacerbation of myoneuropathy, low dose of intravenous hydrocortisone is only recommended in patients in which arterial blood pressure cannot be totally restored with fluid resuscitation and vasopressor therapy (29, 30, 51).

Furthermore, adequate management of sepsis and septic shock in the elderly requires supportive therapy including adequate nutrition, ulcer and deep venous thrombosis prophylaxis, and ventilatory support when necessary. Recent studies have documented that older patients are treated less aggressively than their younger counterparts, particularly those >85 years old (51).

It has generally been argued that aged patients have a significantly shorter life span and that survivors become more dependent and require more social and economical support than other age groups (57). However, in the study of Biston et al. (51) of a cohort of patients with mixed circulatory shock, old patients who survived had a good quality of life a few years after the event.

In the last decades, therapeutic modulation trying to attenuate aberrant immune response during sepsis has been attempted without success (58). Based on evidence that pro- and anti-inflammatory response are mounted simultaneously in septic patients (59), new therapeutic strategies have focused to restore homeostasis of the immune system and boosting immunity to control infection rather than inhibition of the inflammatory response (58–60).

It is important to underline the greater emphasis made on the dysregulated immune response in the last definition of sepsis. Two therapeutic approaches are recently proposed for the modulation of the aberrant immune response to sepsis (Table 2). The extracorporeal blood purification therapies, including convection-based strategies such as high-volume hemofiltration, high cutoff membrane, hemoperfusion with different adsorbents like polymyxin-B or Cytosorb™, and coupled plasma filtration adsorption could potentially have a positive impact in the immune response, despite the effects on removing inflammatory and anti-inflammatory cytokines through a modification of the phenotype and function of immune cells (61). Nevertheless, the efficacy regarding improvement of survival has not yet been elucidated and more clinical studies are needed (61, 62).

**Table 2:** **Immunomodulatory therapies in the septic patient**.

Immune therapies (59, 60, 63–70)	Thymosin alpha-1	Recombinant human interleukin (IL)-7	Granulocyte colony-stimulating factor and granulocity macrophage colony-stimulating factor	Interferon gamma (IFN-γ)	Anakinra	Exogenous immunoglobulins

Possible effect	Produces T-cell and DC maturation and decrease all causes of mortality in septic patients	Improves lymphocyte functionality (CD4 and CD8 T-lymphocyte proliferations, IFN-γ production, or B-cell induction)	Increase neutrophil counts in blood but limited results in sepsis. Improves myelopoeisis and granulopoiesis	Restores expression of HLA-DR in monocytes in all patients. Limited the reduction in the LPS. Induced tumor necrosis factor (TNF)-α response	Blocks interleukin-1, improving survival of patients with sepsis	Antibacterial effect, immunomodulation therapy but not evidence of benefit demonstrated in sepsis
		Decrease Lymphocyte apoptosis	Augments T-cell responses	Reduces infection and related complications		
		Increases IFN-γ secretion				

Extracorporeal blood purification systems (60–62) (no modality confers survival advantage)	Continuos veno-venous hemodialysis with high cutoff dialyzer membranes (HCO)	Hemofiltration/conventional hemofilter	Hemoperfusion	Coupled plasma filtration adsorption		

Possible effect	Effective way to eliminate INF-a, IL-B, IL-2, and IL-6, IL-10, IL-12	Weaker elimination of TNF-α. More efficient in removing soluble receptor for IL-1 compared to continuous HD	Restore HLA-Dr expression on monocytes. Adsorb activated leukocytes and to remove circulating cytokines	Remove inflammatory mediators


The second approach is based on reboosting the immune system (63). Recent studies have shown that HLA-DR expression could be useful in predicting infections and death by identification whether or not a patient with sepsis is in the immunosuppressive phase of the disease. Investigations in this line take relevance when establishing the use of immunomodulatory therapies, and it is possible that some patients may benefit from inhibition immune system response but, on the other hand, other patients could benefit from stimulation of the immune system (58). In this context, monocyte HLA-DR expression could be an optimal marker to determine the most adequate treatment option (59). In this respect, novel therapeutic strategies for severe sepsis and septic shock are currently considered to try to moderate the damage caused by innate immune response in endothelial tissues, and, therefore, to prevent secondary infections o reactivation latent viruses. The use of thymosin alpha 1 has shown very promising results inducing T-cells and dendritic cells maturation and was associated with lower mortality in septic patients (64).

Recombinant human IL-7 demonstrated the ability to restore normal lymphocyte functions, CD4 and CD8 T-lymphocyte proliferation, IFN-γ production, or induction of B-cells after stimulation (65). It has been reported that the administration of IFN-γ to septic patients can restore monocyte HLA-DR expression with the result in clearance of sepsis (66). Additionally, it has been also demonstrated that IFN-γ partially reversed immunoparalysis by attenuating the reduction of TNF-α (67). The use of drugs to increase the number of neutrophils has been also investigated but with limited results. Clinical trials found no evidence supporting the use of granulocyte colony-stimulating factor or granulocity macrophage colony-stimulating factor therapy in reducing mortality rate of septic patients (68). Treatment with the IL-1 blocking agent anakinra has recently showed good results improving survival of patients with organ dysfunction (69).

Therapies with intravenous immunoglobulins have not proven effective in sepsis. The differences in the composition, timing of administration of immunoglobulin in addition to a lack of knowledge of the mechanisms of action could be responsible for the different results obtained in the clinical trials (63, 70).

## Prognosis and Outcome

Elderly patients with severe sepsis and septic shock have high mortality rates of around 50–60%. The mortality rate due to severe sepsis in elderly patients is 1.3–1.5 times higher than in younger cohorts. A number of factors independently associated with death have been identified in critically ill patients, including preinfectious immune or genetic status, nosocomial events, comorbidities, severity of illness, age ≥75 years, and impaired level of consciousness (51, 71). Hospital mortality rates are also higher in frail than in non-frail patients.

Data regarding subsequent survival and quality of life after severe sepsis are limited, especially in the elderly who usually have a poorer functional outcome. The long-term prognosis is mainly dependent on previous functional status rather than on severity of illness at ICU admission (72–75).

It has been suggested that baseline physical function and frailty status could aid in prognosis and informed decision-making for very old critically ill patients (76). The severity of acute disease on admission can also influence in-hospital mortality and mortality after discharge in patients aged 80 or over altering greatly physical function of long-term hospital survivors (77–80).

It has been shown that elderly ICU survivors has a poorer quality of life prior to ICU admission than younger people, and although it could improve after hospital discharge, the quality of life remains lower than that of the general population (78, 79).

In a Canadian cohort study of 610 patients aged 80 years or older who where admitted in the ICU, only 26% of them survived and returned to baseline levels of physical function at 1 year (73). In this study, as lower were age, APACHE II score, Charlson comorbidity index, and frailty index, the physical recovery was greater. Also, the baseline physical function score and the specific diagnoses on admission were associated with recovery (73).

Some recent studies observed that advanced age alone is not a predictive negative factor for the success of surgical and ICU interventions, although associated comorbidities could decrease the likelihood of survival at discharge in all age groups (73, 75–77). It is also known that unplanned admissions to the ICU in elderly patients are associated with poorer outcomes as compared to planned admissions (71, 78).

It has been estimated that up to 29.6% of very patients were refused for ICU treatment, showing a significantly higher survival in ICU-admitted octogenarians than for patients who were considered too ill or old for ICU admission (71–77).

## Conclusion

Management of sepsis is a frequent ICU challenge, with substantial mortality and a significant consumption of health-care resources. It is estimated that the number of cases of severe sepsis will grow at a rate of 1.5% per year, with a proportional increase in the group of patients with advanced age. The increased incidence of sepsis in elderly people is associated with a number of predisposing factors, including comorbid diseases, preadmission status, malnutrition, frailty, and an impaired function of the immune system (the so-called immunosenescence). The immunosenescence status increases both the risk for sepsis and a more severe presentation of infection and is probably related with a poor outcome.

Different studies have shown that older people have a higher mortality in association with severe sepsis, but it is important for clinicians to consider separately the general prognosis of a population of older patients and the prognosis of the individual patient. Age as a single factor predicts the length of stay in the ICU, but should not be used either to estimate the outcome or to determine treatment options for individual patients, given that the increased mortality observed in older patients with severe sepsis is thought to be due to the increased comorbidities among this group.

Differences in treatment decisions regarding the use of more aggressive treatment based only on age without considering other prognostic factors could lead an important number of older patients to prevent from the benefit from such treatment. On the other hand, although survival is the most important outcome, the quality of life of survivors is also relevant. New therapeutic approaches trying to handle aberrant immune responses like immunomodulatory therapies are emerging and may be the future in the management of infection, particularly in older patients. A better immunologic characterization of the patient is a cornerstone to get a better modulatory therapy of the immune response.

Elderly population will increase in the forthcoming years, so it is a priority for clinicians, especially intensivists, to understand the extent of the problem, socially and economically, and to assess therapeutic challenges and outcomes from a more efficient and effective way.

## Author Contributions

All authors contributed equally to the conception and design of the study and to the drafting of the article.

## Conflict of Interest Statement

The authors declare that the research was conducted in the absence of any commercial or financial relationships that could be construed as a potential conflict of interest.
